# Screening of the Open Source Malaria Box Reveals an Early Lead Compound for the Treatment of Alveolar Echinococcosis

**DOI:** 10.1371/journal.pntd.0004535

**Published:** 2016-03-11

**Authors:** Britta Stadelmann, Reto Rufener, Denise Aeschbacher, Markus Spiliotis, Bruno Gottstein, Andrew Hemphill

**Affiliations:** Institute of Parasitology, Vetsuisse Faculty, University of Berne, Berne, Switzerland; Drexel University, UNITED STATES

## Abstract

The metacestode (larval) stage of the tapeworm *Echinococcus multilocularis* causes alveolar echinococcosis (AE), a very severe and in many cases incurable disease. To date, benzimidazoles such as albendazole and mebendazole are the only approved chemotherapeutical treatment options. Benzimidazoles inhibit metacestode proliferation, but do not act parasiticidal. Thus, benzimidazoles have to be taken a lifelong, can cause adverse side effects such as hepatotoxicity, and are ineffective in some patients. We here describe a newly developed screening cascade for the evaluation of the *in vitro* efficacy of new compounds that includes assessment of parasiticidal activity. The Malaria Box from Medicines for Malaria Venture (MMV), comprised of 400 commercially available chemicals that show *in vitro* activity against *Plasmodium falciparum*, was repurposed. Primary screening was carried out at 10 μM by employing the previously described PGI assay, and resulted in the identification of 24 compounds that caused physical damage in metacestodes. Seven out of these 24 drugs were also active at 1 μM. Dose-response assays revealed that only 2 compounds, namely MMV665807 and MMV665794, exhibited an EC_50_ value below 5 μM. Assessments using human foreskin fibroblasts and Reuber rat hepatoma cells showed that the salicylanilide MMV665807 was less toxic for these two mammalian cell lines than for metacestodes. The parasiticidal activity of MMV665807 was then confirmed using isolated germinal layer cell cultures as well as metacestode vesicles by employing viability assays, and its effect on metacestodes was morphologically evaluated by electron microscopy. However, both oral and intraperitoneal application of MMV665807 to mice experimentally infected with *E*. *multilocularis* metacestodes did not result in any reduction of the parasite load.

## Author Summary

The fox tapeworm *Echinococcus multilocularis* causes a deadly disease in humans that is characterized by cancer-like parasite growth in the liver of patients. Hence *E*. *multilocularis* is ideally removed by radical resection. However, as this is not always possible, patients are treated by chemotherapy to stop further parasite growth. To date, the drugs in use cannot kill the parasite *E*. *multilocularis* and they also induce side-effects, therefore new options for treatment are needed. We here screened a library of 400 compounds with proven activity against *Plasmodium falciparum*, the cause of malaria, for their effects against *E*. *multilocularis in vitro*. We developed and refined an *in vitro* screening cascade and developed an assay that can identify compounds with parasiticidal effects against *E*. *multilocularis* cells. We here show that MMV665807 acts parasiticidal as demonstrated by *in vitro* viability tests and by electron microscopy. In the mouse infection model the drug was not active, but work will focus on the evaluation of derivatives with improved bioavailability and pharmacokinetic properties to achieve parasiticidal activity not only *in vitro*, but also *in vivo*.

## Introduction

The metacestode stages of the two cestode species *Echinococcus granulosus sensu lato* and *E*. *multilocularis* cause cystic echinococcosis (CE) and alveolar echinococcosis (AE), respectively. Worldwide, CE is more abundant and accounts for more than 1 million DALYs (disability-adjusted life years) in humans, while AE is restricted to the Northern hemisphere and has been estimated to cause more than 600,000 DALYs per year [1], an impact that is largely underreported [2], but nevertheless comparable to other neglected tropical diseases such as trypanosomiasis, Chagas disease, and schistosomiasis [1].

The adult hermaphroditic tapeworms persist in the intestine of their final hosts (mainly dogs for *E*. *granulosus s*.*l*. and mainly foxes for *E*. *multilocularis*), and produce eggs that are released into the environment with the faeces. Accidental uptake of eggs by a variety of intermediate host species, including humans, leads to the activation and release of the first larval stage, the oncosphere, which then penetrates the intestinal wall, is disseminated via blood and lymphatic vessels, and finally forms the subsequently maturating larval stage, the metacestode, mainly in the liver of infected individuals. This disease-causing metacestode exhibits tumor-like properties for AE. In the case of CE, it forms a large and well delineated unilocular and fluid-filled cyst that steadily increases in size, whereas in AE the metacestodes grow by asexual budding of vesicles that spread infiltratively into surrounding organs and form a multivesicular lesion. This renders *E*. *multilocularis* the most deadly of all helminth infections. Complete surgical removal would in theory be the best treatment option for both diseases, but it in reality, clinical management of CE and AE is very complex (for a more comprehensive overview see guidelines given in [3]). Inoperable cases are treated by chemotherapy, which is currently based on the application of the benzimidazoles albendazole (ABZ) and mebendazole (MBZ). In any case, surgery is also accompanied by benzimidazole treatment [4,5]. Although these drugs have improved the life-expectancy of patients considerably, they are not effective in all cases, and cause toxicity in a considerable number of patients leading to discontinuation of treatment [6]. Furthermore, in the case of AE, these drugs only block further metacestode growth and do not act parasiticidal. Thus, alternative, and preferentially better, treatment options are needed.

A major advancement in *Echinococcus* research has been the development of an *in vitro* culture technique for the maintenance and efficient propagation of fully infective *E*. *multilocularis* metacestodes [7–9]. Subsequently, this culture system enabled researchers to isolate and culture stem cells and to establish RNAi technology [9,10]. More recently, sequencing of the genomes of *E*. *multilocularis* and of *E*. *granulosus s*.*l*., and publication of transcriptome data during different stages of their life cycle [11,12] has provided a plethora of data that is openly accessible and can be exploited for the identification of metabolic pathways and characterization of novel drugs and drug targets. By employing a drug screening assay that is based on the release of phosphoglucose isomerase (PGI) by physically damaged *E*. *multilocularis* metacestodes [13], a variety of novel drugs and drug classes have been investigated, and several interesting compounds with profound *in vitro* and *in vivo* activity were identified (for review see [6]). However, AE and CE have not attracted the attention of pharmaceutical companies yet, since the market has not been properly assessed, as there is a high number of asymptomatic carriers and a long latency period before outbreak of the disease [2]. Thus to date, pharmaceutical industry does not provide the incentive to develop novel drugs due to the horrendous costs associated with the drug development process. Another strategy is to focus on already described drugs and drug classes that were originally developed for other diseases [6,7,14]. In this context, we have recently screened a commercially available Food and Drug Administration (FDA) approved drug library (426 compounds), and identified the proteasome as a potential drug target in cestodes [15]. A slightly different approach, focusing on anti-infective agents, showed that anti-malarial compounds such as artemisinin and derivatives, as well as mefloquine, have anti-echinococcal effects in *vitro* and *in vivo* [16–19]. Based on these previous findings we here report on the screening of the Malaria Box from Medicines for Malaria Venture (MMV). The open access Malaria Box includes 200 drug-like and 200 probe-like compounds, which represent a subset of the 20,000 anti-malarials identified *in vitro* from the high-throughput screening efforts of St. Jude Children's Research Hospital (TN, USA), Novartis and GlaxoSmithKline [20–22] that were selected for commercial availability, oral bioavailability and chemical diversity [23].

We here present a detailed *in vitro* screening cascade that was applied for screening of the Malaria Box against *in vitro* cultured *E*. *multilocularis* metacestodes. In addition to the previously described PGI assay [13] and measurement of host cell toxicity, further *in vitro* assays addressed the parasiticidal potential of compounds. One compound, MMV665807 was evaluated in two *in vivo* mouse models for its activity in mice infected by intraperitoneal injection of *E*. *multilocularis* metacestodes.

## Materials and Methods

If not stated otherwise, all chemicals were purchased from Sigma (St. Louis, MO, USA). Dulbecco’s modified Eagle medium (DMEM) and fetal bovine serum (FBS) were from Biochrom (Berlin, Germany), and all other cell culture reagents were from Gibco-BRL (Zürich, Switzerland). The open-source Malaria Box containing 400 chemical compounds was supplied by Medicines for Malaria Venture (MMV, Geneva, Switzerland) in five 96 well plates, prepared as 10 mM stock solutions in DMSO [23]. The drugs MMV665807 and MMV665794 were obtained through Princeton Bio Molecular Research (Monmouth Junction, NJ, USA) and Specs (Zoetermeer, Netherlands).

### *In vitro* culture and drug screening against *E*. *multilocularis* metacestodes

The preparation of *E*. *multilocularis* metacestode material (strain H95) from intraperitoneally (i.p.) infected BALB/c mice was performed as described earlier [8]. Metacestode vesicles were used for *in vitro* drug screening when they reached 2 to 4 mm in diameter [13].

For screening of the Malaria Box against *E*. *multilocularis* metacestodes, the PGI screening assay was applied that measures the release of the enzyme phosphoglucose isomerase (PGI) upon physical impairment of metacestodes [13]. The screening setup was done in 24 well plates as described by Stadelmann et al [13]. For initial screening, each drug was applied at 10 μM to metacestodes in single wells. As a positive control-drug, the dicationic diguanidino compound DB1127 (10 μM) was used [19,24] and 0.1% DMSO was added as negative control. Metacestodes were cultured in the presence of the compounds for 5 days, after which PGI release was quantified exactly as stated in Stadelmann et al [15]. Subsequently, a secondary screening at 1 μM was performed in triplicates including those compounds that had exerted at least 50% PGI release in relation to DB1127. Those compounds that also exhibited activity at 1 μM were further subjected to a dose-response study at concentrations of 10, 5, 1, 0.5, 0.1, 0.05 and 0.01 μM, all in three independent setups. Averages, standard deviations and EC_50_ values were calculated in Microsoft Excel 2010.

### Assessment of *in vitro* toxicity in human foreskin fibroblasts and Reuber rat hepatoma cells

The toxicity for human foreskin fibroblasts (HFF) and Reuber rat hepatoma (RH) cells was assessed *in vitro* for the most active drugs of the PGI screen. For testing growth inhibitory effects on confluent host cells, cells were seeded in 96-well plates (10,000 cells per well for HFF and 50,000 cells per well for RH cells), in DMEM containing FBS (10%) and antibiotics (100 U penicillin, 100 μg streptomycin and 0.25 μg amphotericin B per mL medium) and were cultured overnight at 37°C, 5% CO_2_. Thereafter the drugs (10 μM) were added and diluted in a serial 1:2 dilutions down to 20 nM. The concentration range was further adjusted in subsequent setups. For testing growth inhibitory effects on proliferating cells, cells were seeded at 1,000 (HFF) and 5,000 (RH) cells per well, and were allowed to attach for 5 hours at 37°C before addition of the drugs. After 5 days of culture, cell viability was assessed by alamarBlue assay [25]. For this, resazurin was added to a final concentration of 10 mg/L to each well and fluorescence measured after 0 and 3 h of incubation in the EnSpire multilabel reader (Perkin Elmer, Waltham, MA, USA). IC_50_ values were calculated in Microsoft Excel 2010 after logit-log transformation and averages and standard deviations of three independent setups were calculated.

### Assessment of *in vitro* toxicity in *E*. *multilocularis* germinal layer cell cultures

To assess the effects of MMV665807 on the viability of parasite cells, germinal layer cells were extracted according to Spiliotis et al [9]. After overnight aggregate formation, 15 units of cells were distributed to a black 384 well plate (Nunc, Thermo Scientific, Reinach, Switzerland) in 12.5 μL medium. A concentration series (final concentrations of 3, 1, 0.3, 0.1, 0.03, 0.01 μM) of MMV665807, MMV665794 and mefloquine were prepared in medium and added 1:2 to the cells. For mefloquine, the concentration range was adjusted in a subsequent setup up to 100 μM. As negative control, DMSO was added accordingly. After 5 days of culture at 37°C under humid nitrogen atmosphere, 25 μL CellTiter-Glo (Promega, Dübendorf, Switzerland) including 1% TritonX-100 was added. Plates were incubated for 15 minutes on a shaker at room temperature and complete disruption of all cell aggregates was checked for before measurement of luminescence on a 2300 Enspire multilabel reader (Perkin-Elmer). Mean values and standard deviations were calculated and DMSO values set to 100% viability. The IC_50_ value was calculated in Microsoft Excel 2010 after logit-log transformation and averages as well as standard deviations of four independent setups were calculated.

### Assessment of metacestode viability by alamarBlue assay

The viability of the metacestode tissue was assessed by alamarBlue assay. The general setup of vesicles and drugs was the same as for the PGI assay, but MMV665807 and mefloquine were applied in a concentration series ranging from 100 to 0.1 μM using 1:2 dilution steps. After 12 days of culture in the presence of the drugs, metacestodes were disrupted by mechanical breaking with a 1 mL pipette tip. Subsequently, resazurin was added to 20 mg/mL final concentration from a 200x stock and mixed well with the disrupted metacestodes. Fluorescence was measured after 0 and 5 h of incubation in the EnSpire multilabel reader (Perkin Elmer). The relative increase in fluorescence and respective IC_50_ values were calculated in Microsoft Excel 2010 after logit-log transformation. The experiment was performed three times independently and the average and standard deviations were calculated in Microsoft Excel 2010.

### Transmission electron microscopy

*In vitro-*cultured metacestodes were cultured for five days in the presence of 1.6, 0.8, 0.4 or 0.1 μM MMV665807, or in the absence of the drug, and were processed for TEM as previously described [15]. In short, primary fixation was carried out in 2% glutaraldehyde in 0.1 M sodium-cacodylate buffer, pH 7.3 for 1 h, and post-fixation in 2% osmium tetroxide in 0.1 M sodium-cacodylate buffer for 2 h. Samples were then pre-stained in saturated uranyl acetate solution in water for 30 min, washed in water, and were stepwise dehydrated in ethanol (30%, 50%, 70%, 90%, and three times 100%). Subsequently specimens were embedded in epoxy resin and polymerization of the resin was carried out at 60°C for 12 h. Ultrathin sections were cut using an ultramicrotome (Reichert and Jung, Vienna, Austria), and were loaded onto formvar-carbon coated nickel grids (Plano GmbH, Marburg, Germany). Specimens were stained with uranyl acetate and lead citrate, and were viewed on a Phillips EM400 transmission electron microscope operating at 80 kV.

### Ethics statement

The *in vivo* studies were performed in compliance with the Swiss animal protection law (TschV, SR 455). The study was approved by the Animal Welfare Committee of the Canton of Berne (license numbers BE 103/11 and BE 112/14).

### *In vivo* assessment of MMV665807 treatment in *E*. *multilocularis* infected mice

Animals were kept at a temperature-controlled 12/12 hours light cycle with food and water *ad libitum* and according to the Swiss Animal protection law. For the *in vivo* treatment of MMV665807, BALB/c mice (from Charles River, Sulzfeld, Germany), at the age of 8 weeks with an average body weight of 20 g, were i.p. infected with 200 μL metacestodes vesicle suspension. This material was prepared from *in vitro*-cultured metacestodes (strain H95), which were disintegrated by pressing the parasites through a 1 mL pipette tip. Parasite tissue was centrifuged at 500 x g for 5 min at room temperature before being mixed 1:1 in PBS.

Peroral (p.o.) drug treatments by gavage were initiated 6 weeks post-infection and performed as follows: group 1 received no treatment at all; group 2 received 100 μL corn oil containing 7.8 μL DMSO; group 3 mice were treated with 100 μL corn oil containing albendazole (ABZ) in 7.8 μL DMSO, in order to obtain a dosage of 200 mg/kg body weight (positive control); group 4 was treated with 100 μL corn oil containing MMV665807 in 7.8 μL DMSO, resulting in 100 mg/kg body weight. All mice were treated for a total time period of 4 weeks, with consecutive treatment during 5 days per week, and interruption of treatment for 2 days each week. At the end of the treatment, all mice were sedated by isoflurane and subsequently euthanized by CO_2_. All parasite tissue was resected, placed into a petri dish, and the parasite mass was weighed. Statistical analysis of the *in vivo* experiments was performed in R version 3.0.1. The data distribution was checked by Shapiro-Wilk test and eventual differences between treatment groups identified by the non-parametric Kruskal-Wallis test. P values were calculated by the non-parametric Wilcoxon signed-rank test with Bonferroni-adjustment. Boxplot visualizations of the data were prepared in Microsoft Excel 2010.

I.p. drug treatments were initiated 2 weeks post-infection. 32 infected mice were randomly separated into 4 groups with 2 times 4 animals per cage. All animals were exposed to 4 weeks of treatment, with five consecutive days of drug application followed by a 2 day recovery period without treatment. Treatment groups were defined as follows: group 1 (control) received 50 μL of corn oil by gavage on 5 days per week, and an i.p. injection of DMSO in corn oil (4 μL DMSO and 46 μL corn oil) on three days per week; group 2 also received corn oil by gavage 5 days per week, and an i.p. injection of MMV665807 (100 mg/kg in 4 μL DMSO plus 46 μL corn oil) three times per week; group 3 was treated with the standard albendazole treatment (albendazole in corn oil, 200 mg/kg/ day for 5 days each week) and also received an i.p. injection of 4 μl DMSO suspended in 46 μl corn oil three times per week; group 4 received a combined treatment consisting of albendazole in corn oil by gavage (200 mg/kg/day 5 days per week) and an i.p. injection of MMV665807 (100 mg/kg three times per week). After 4 weeks of treatment, mice were euthanized and the parasite mass resected and measured as described above. Statistical analyses were performed as described above.

## Results

### *In vitro* screening of the Malaria Box identifies a compound with selective toxicity against *E*. *multilocularis* metacestodes and isolated germinal layer cells

**Fig 1:**
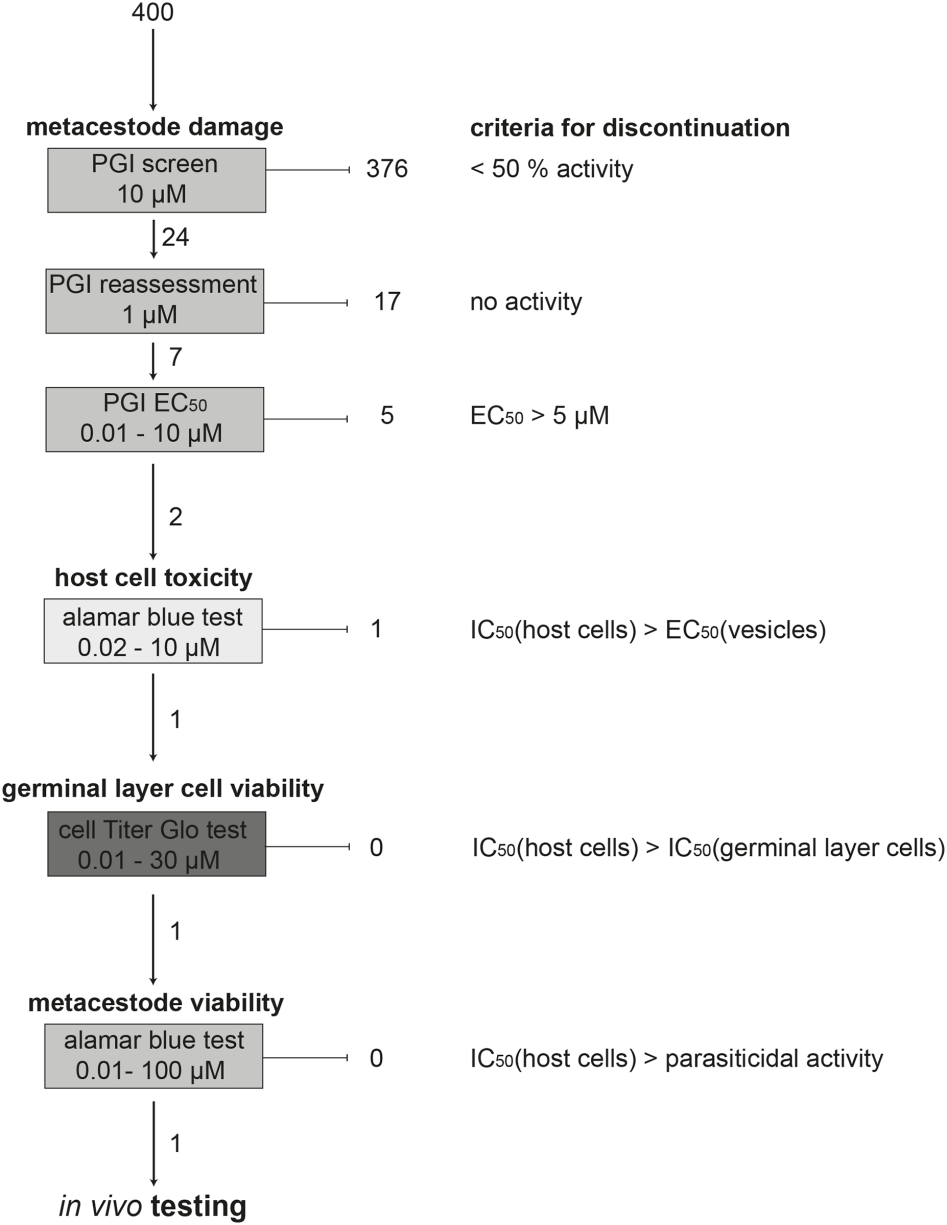
Screening cascade for *in vitro* assessment of drug activity against *E*. *multilocularis* metacestodes. A total of 400 compounds of the Malaria Box were tested first for their ability to induce metacestode damage, employing the PGI assay in singlets at 10 μM. Compounds that showed less than 50% activity of the control were discontinued. 24 active drugs were re-assessed at 1 μM in triplicates. 17 compounds were shown to be inactive and 7 compounds were followed up (see Fig 2). The EC_50_ values of these drugs was determined by PGI assay in triplicates (10–0.01 μM) and drugs with an EC_50_ value of more than 5 μM were discontinued. In a second major step, host cell toxicity against Reuber rat hepatoma (RH) cells and human foreskin fibroblasts (HFF), both either at a confluent and proliferative state, was assessed by alamarBlue test, and only drugs with a potential therapeutic window were continued. For the remaining MMV665807 the toxicity for isolated and cultured germinal layer cells was assessed (30–0.01 μM) and the IC_50_ was determined. Since the IC_50_ against host cells was higher than against germinal layer cells of *E*. *multilocularis*, the drug was tested for its ability to reduce the viability of metacestodes by alamarBlue test (see Fig 3). The drug MMV665807 that still showed parasiticidal activity at concentrations below the host cell toxicity, finally entered *in vivo* testing (see Fig 7).

**Fig 2:**
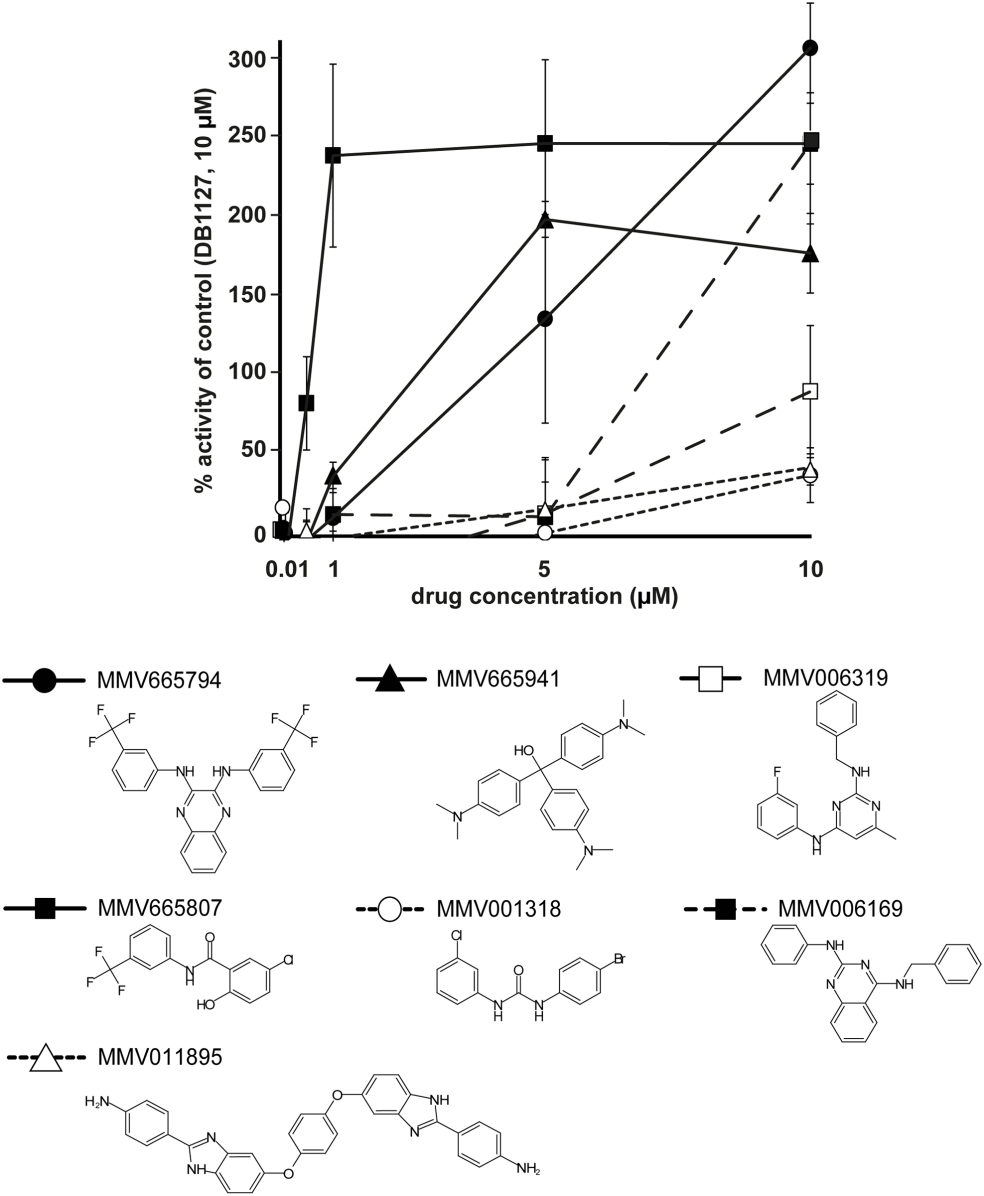
Drug concentration series of seven compounds against *E*. *multilocularis* metacestodes. Displayed are compounds that exhibited more than 50% activity of the control DB1127 in an initial screen at 10 μM and also activity at 1 μM in the PGI assay against *E*. *multilocularis* metacestodes: MMV665794, MMV665941, MMV006319, MMV665807, MMV001318, MMV006169 and MMV011895. Their structures are given below the graph. The seven compounds were tested by PGI assay on *E*. *multilocularis* metacestodes in a concentration series ranging from 0.01 to 10 μM in triplicates. DB1127 (10 μM) served as a positive control and was set as 100%. DMSO was the internal negative control that was subtracted from all other values. Note the high activity of MMV665807 down to low concentrations.

In a primary screen, the open access MMV library of 400 anti-malarial compounds was screened for activity against *E*. *multilocularis* metacestodes employing a concentration of 10 μM (Fig 1). Physical damage upon drug exposure was evaluated by PGI assay. The full list of tested drugs is shown in S1 Table. DB1127, a dicationic diguanidino compound shown to be active against metacestodes *in vitro* and *in vivo* [26], was used as a positive control. Results are presented in relation to the activity of DB1127. 24 compounds exhibited more than 50% activity compared to DB1127. These 24 compounds were further assessed at 1 μM, and seven compounds exhibited clear effects, and were therefore further analyzed in a dose-response experiment using concentrations from 10 to 0.01 μM (Fig 2). According to this concentration series, two compounds showed clear anti-metacestode activity also at lower concentrations: the salicylanilide MMV665807 (CHEMBL589733) and MMV665794 (TCMDC-124162, for structures see Fig 2). The EC_50_ values of these compounds against *E*. *multilocularis* metacestodes, as determined by PGI assay, were calculated to be 1.2 and 2.8 μM respectively (Table 1).

**Table 1:** *In vitro* activities of MMV665794 and MMV665807 against *E*. *multilocularis* metacestodes, germinal layer cells, HFF and RH cells.

	MMV665807	MMV665794	DB1127	Mefloquine
**Confluent HFF (IC**_**50**_**)**	29.2 ± 0.06	21.5 ± 0.17	3.2^(1)^	ND
**Preconfluent HFF (IC**_**50**_**)**	4.8 ± 0.59	5.9 ± 0.21	ND	ND
**Confluent RH (IC**_**50**_**)**	20.5 ± 0.13	0.8 ± 0.15	3.6^(1)^	ND
**Preconfluent RH (IC**_**50**_**)**	4.3 ± 0.14	1.1 ± 0.18	ND	ND
***E*.*m*. metacestodes (EC**_**50**_**)**	1.2 ± 1.6	2.8 ± 0.53	6.1^(1)^	> 30^(2)^
***E*.*m*. GL cells (IC**_**50**_**)**	0.6 ± 0.37	5.8 ± 0.16	ND	13.8 ±0.33

*In vitro* activities of MMV665794 and MMV665807 (in μM) against *E*. *multilocularis* metacestodes, germinal layer (GL) cells, HFF and RH cells, in comparison to previously characterized compounds DB1127 and mefloquine. Viability measurements on HFF and RH cells employed alamarBlue assay, while for *E*. *multilocularis* metacestodes the physical damage imposed upon drug exposure was measured by PGI assay and for *E*. *multilocularis* germinal layer cells viability was assessed by CellTiter-Glo assay. Average values of three independent measurements are given with the respective standard deviation.

^(1)^ data obtained from [26];

^(2)^ data obtained from [19]; ND = not determined.

MMV665807 and MMV665794 were assessed for their effects on the viability of isolated *E*. *multilocularis* germinal layer cells by CellTiter-Glo assay (Fig 1). The IC_50_ value of MMV665807 was 0.6 μM, while MMV665794 was clearly less effective (IC_50_ = 5.8 μM). Mefloquine, also shown previously to be active against *E*. *multilocularis in vitro* and *in vivo* [16,17] was used here for comparison and showed a much lower activity (IC_50_ = 13.8 μM, see Table 1). This confirmed the excellent anti-parasitic activity of MMV665807.

### Effects of MMV665794 and MMV665807 on the viability of human foreskin fibroblasts (HFF), and Reuber rat hepatoma (RH) cells

The viability of HFF and RH cells was assessed using confluent and pre-confluent cultures of HFF and RH cells employing the alamarBlue viability assay (Fig 1). The corresponding IC_50_ values are shown in Table 1. In terms of a potential therapeutic window, MMV665794 exhibited less favorable properties, as the drug was toxic for HFF and RH cells in the same concentration range as for *E*. *multilocularis* germinal layer cells. In contrast, MMV665807 exhibited less toxicity against mammalian cell cultures compared to germinal layer cells, and thus remained as the most promising compound from the Malaria Box (Fig 1).

### A novel *in vitro* viability assay for *E*. *multilocularis* metacestodes

**Fig 3:**
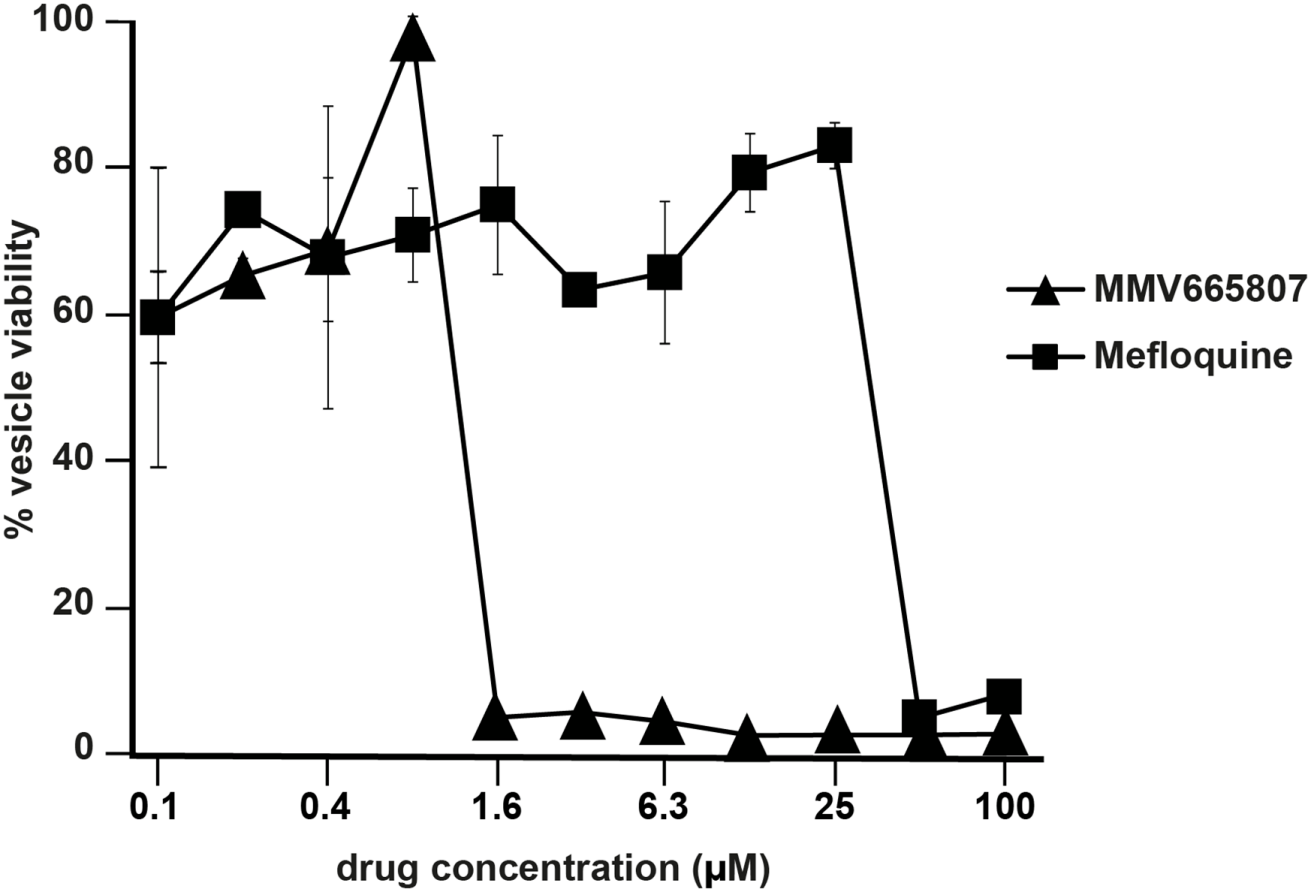
AlamarBlue vesicle viability assay. The vesicle viability assay was developed to assess the potential of drugs to kill cells residing within *E*. *multilocularis* metacestode vesicles, thus being potentially parasiticidal. Vesicle viability was assessed by alamarBlue assay in triplicates. The drug solvent DMSO served as an internal negative control and was set to 100%. Mefloquine was applied since it is known that this drug is not fully parasiticidal [17] and it reduced the vesicle viability only at concentrations of 50 μM or higher. The compound MMV665807 was killing cells within vesicles at concentrations as low as 1.6 μM. This underlines the potential parasiticidal activity of the drug MMV665807. Note that the figure depicts average values obtained from three independent measurements and respective standard deviations are shown. All values and SDs are provided in S2 Table.

Since no drug with true parasiticidal activity against *E*. *multilocularis* has been identified so far, a viability test for the measurement of drug sensitivity was developed that can be applied on whole metacestodes. Viability measurements of intact metacestodes are also based on resazurin-reduction and are done on parasite tissue that is still residing within the vesicles. MMV665807 and mefloquine were comparatively assessed. The assay showed that MMV665807 had the potential to almost completely abolish the viability of the metacestode tissue at concentrations as low as 1.6 μM, whereas mefloquine could only do so at 50 μM or higher (Fig 3).

### Electron-microscopical visualization of the effects of MMV665807 treatment in *E*. *multilocularis* metacestodes

**Fig 4:**
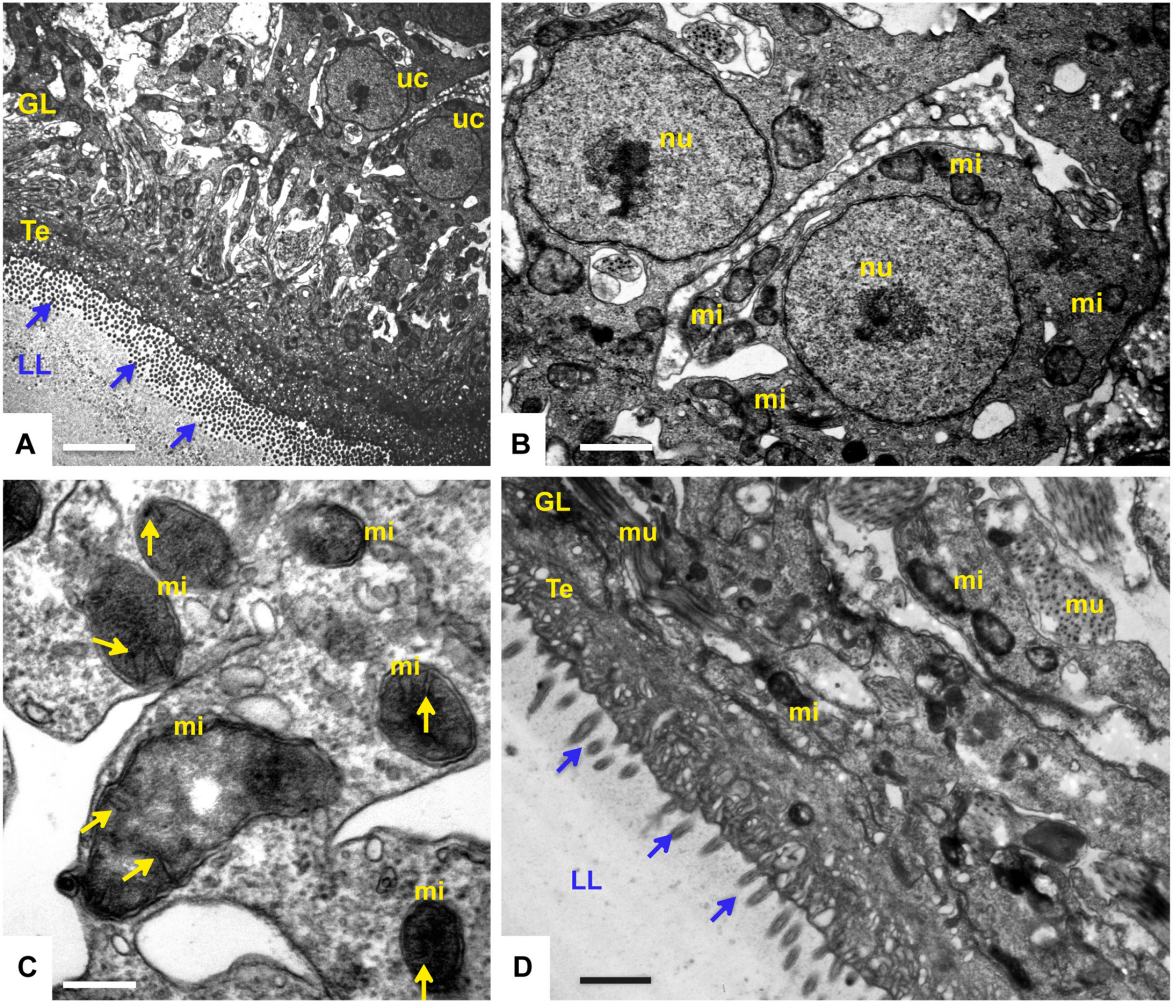
TEM of *E*. *multilocularis* metacestodes: Non-treated or treated with 0.4 μM MMV665807. Non-treated metacestodes (A-C) or metacestodes treated with 0.4 μM MMV665807 for 5 days (D) are shown. (A) is a low magnification view of a section through a non-treated metacestode wall, showing the laminated layer (LL), tegument (Te) and the germinal layer (GL). Clearly visible are undifferentiated stem cells (uc) and the microtriches protruding from the tegument well into the LL (arrows). Bar = 4 μm. Undifferentiated stem cells are shown at higher magnification in (B). mi = mitochondria; nu = nucleus with nucleolus. Bar = 1.5 μm. Higher magnification view of mitochondria are shown in (C). mi = mitochondria, yellow arrows point towards the cristae embedded in an electron matrix. Bar = 0.3 μm. (D) shows a representative micrograph of a metacestode exposed to 0.4 μM MMV665807 for 5 days. Arrows point towards microtriches, mi = mitochondria, mu = muscle cell. Bar = 2.2 μm.

**Fig 5:**
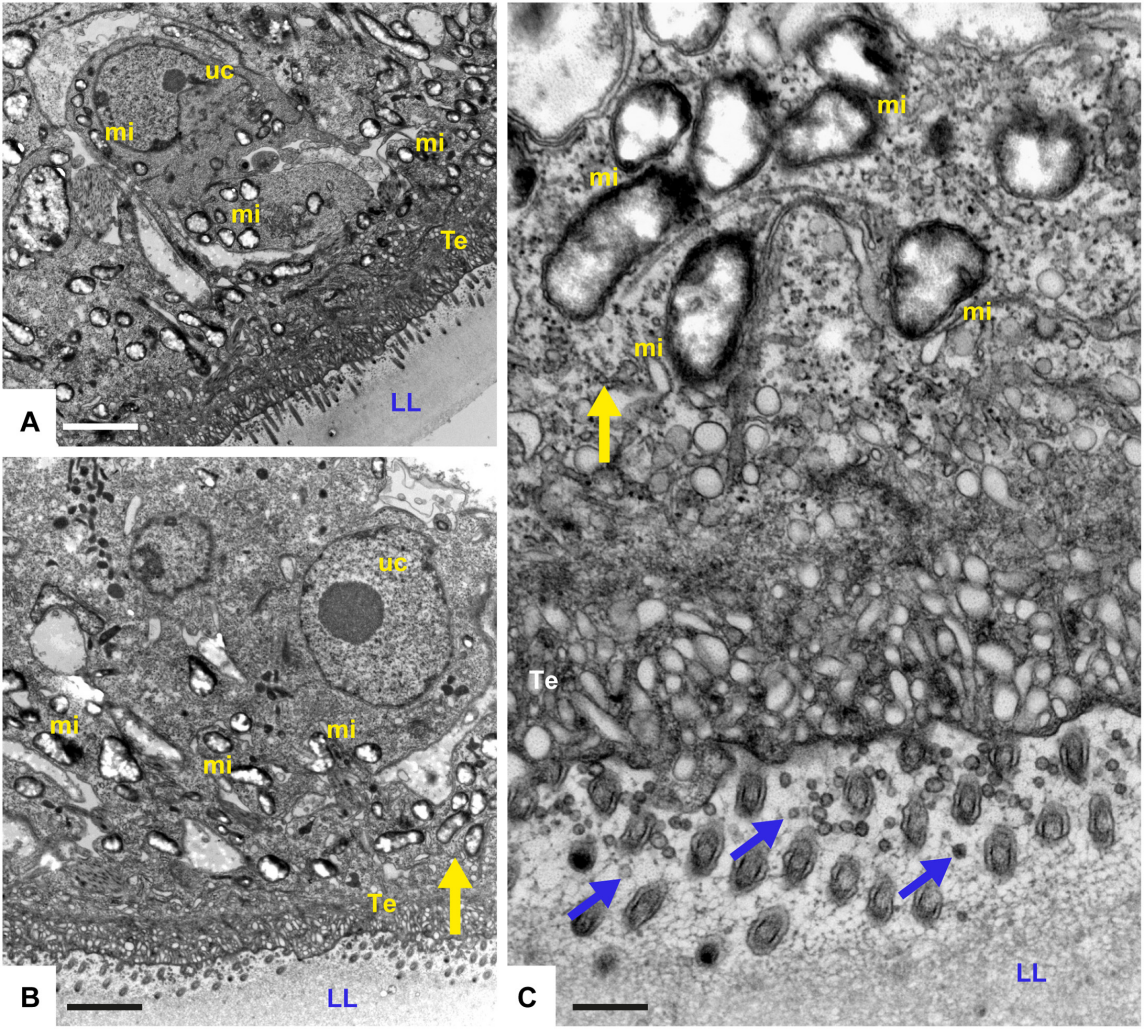
TEM of *E*. *multilocularis* metacestode exposed to 0.8 μM MMV665807 for 5 days. (A) and (B) are lower magnification views. LL = laminated layer, Te = tegument, uc = stem cell with large nucleus and nucleolus. Note largely translucent mitochondria in drug treated parasites (mi). Bars in (A) and (B) = 3 μm. (C) = higher magnification view of mitochondria lacking any clearly discernible internal structures. Also note the presence of clearly discernible accumulation of small vesicles within the LL in the close vicinity of the microtriches (arrows). The yellow arrows in (B) and (C) point towards the same location. Bar = 0.3 μm.

**Fig 6:**
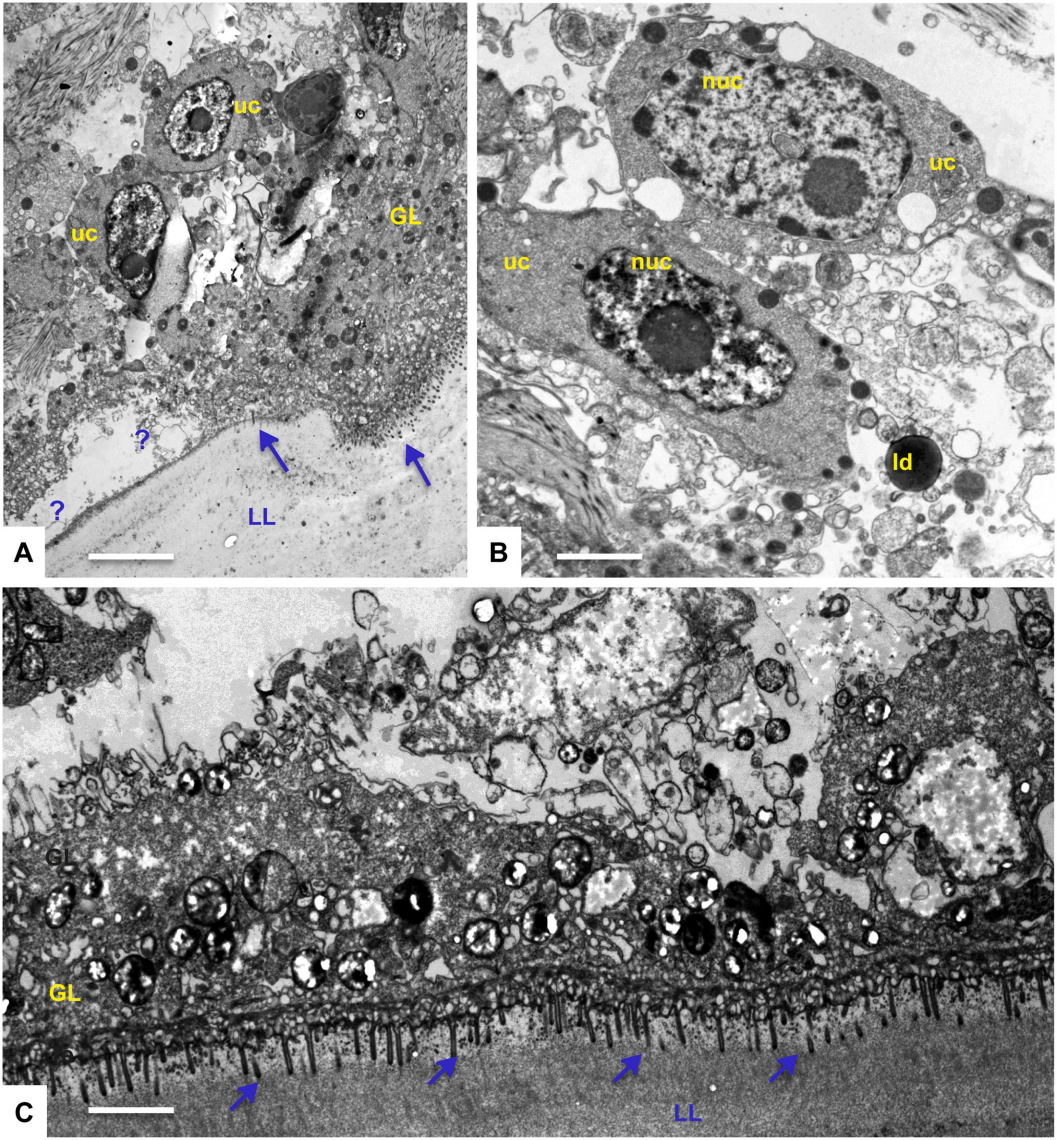
TEM of *E*. *multilocularis* metacestode exposed to 1.6 μM MMV665807 for 5 days. Largely non-viable metacestode tissue is seen. (A) shows the separation of the laminated layer (LL) from the tegument (Te) (marked with +), and undifferentiated cells (uc) with a nucleus that is largely consisting of condensed chromatin. Arrows in (A) point towards microtriches, ld in (B) = lipid droplet. (C) shows a metacestode tissue exhibiting large scale necrosis, with the cytoplasm of the tegument being filled with large and small vacuoles of differing content. Arrows in (C) point towards microtriches. Bars in (A) = 4 μm, (B) = 1.5 μm, (C) = 3 μm.

Metacestodes cultured during a period of 5 days in the presence of solvent (0.1% DMSO) are shown in Fig 4A–4C. The parasite tissue is composed of an outer, acellular laminated layer, which is rich in carbohydrates and separates the live parasite tissue from the environment. Attached to the inner surface of the laminated layer is the tegument, a syncytial structure that exhibits numerous microtriches protruding well into the laminated layer. The inner lining of the tegument is covered by the germinal layer, which contains numerous cell types including muscle cells, subtegumentary cytons, connective tissue and undifferentiated stem cells, which are characterized by a large nucleus and nucleolus (Fig 4B). These cells contain numerous mitochondria, which are filled with an electron dense matrix and cristae, similar to mitochondria in mammalian cells (Fig 4C). Metacestodes treated with 0.1 μM and 0.4 μM MMV665807 did not show any major alterations, and a representative micrograph is shown in Fig 4D. Clear alterations in the *Echinococcus* ultrastructure were detected in parasites treated with 0.8 μM MMV665807 (Fig 5). Alterations were mostly evident in the mitochondrial matrix, which had lost its electron-dense appearance, and cristae were not detectable anymore. Nevertheless, the outer mitochondrial membranes were still evident. Other components of the germinal layer and the tegument appeared less affected, although the laminated layer harbored an increased number of small vesicles in the vicinity of the microtriches, seemingly budding off from these tegumental protrusions. Effects of the drug were most evident at a concentration of 1.6 μM, and clear signs of cell death were found (Fig 6). These included extensive chromatin condensation in the nuclei of germinal layer cells, partial separation of the tegument from the laminated layer, and accumulation of lipid droplets. The germinal layer cells had disintegrated and the tegumental layer was often filled with vacuoles, some of which exhibiting electron dense content of unknown nature. Thus, treatment with 1.6 μM MMV665807 rendered metacestodes largely non-viable.

### Peroral treatment of secondary infected mice

**Fig 7:**
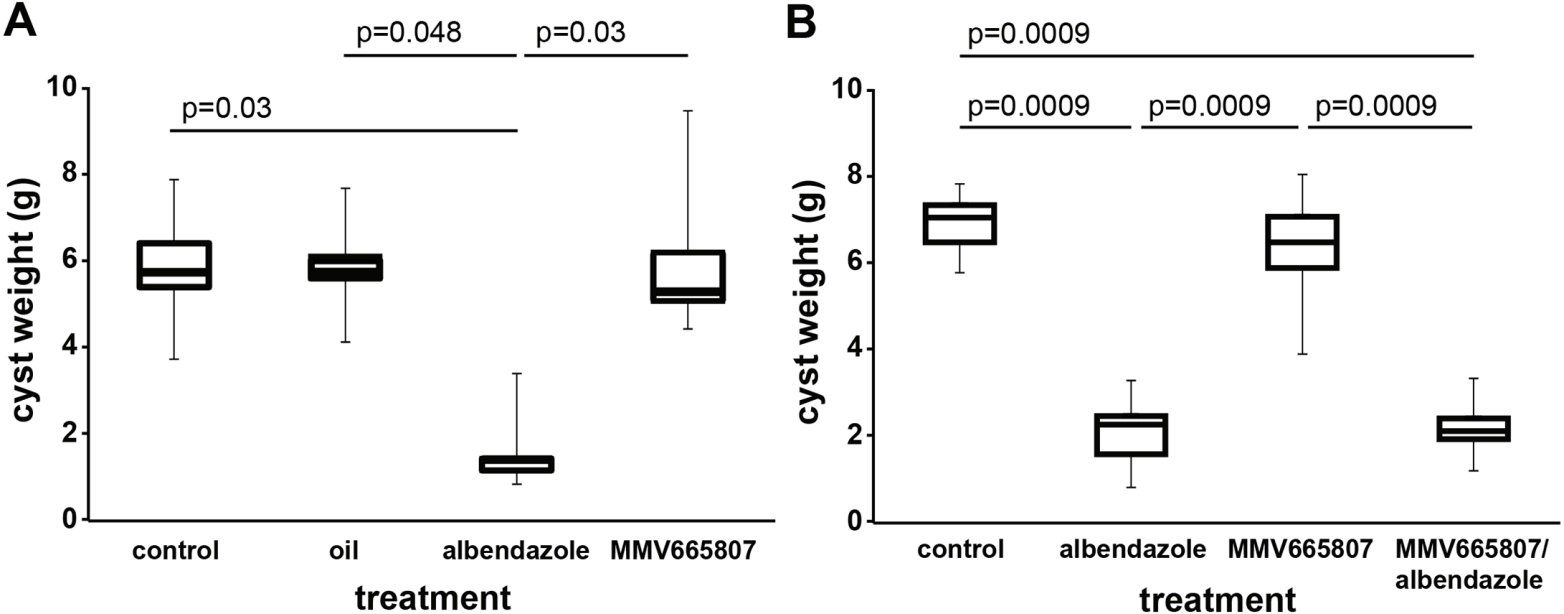
*In vivo* MMV665807 treatment in the secondary alveolar echinococcosis mouse model. (A) Balb/c mice were i.p. infected with metacestodes obtained from *in vitro* cultures. After 6 weeks of infection, mice were randomly allocated into 4 groups of 6 mice and treated p.o. during 4 weeks. The different treatment groups were: control (no treatment), oil (p.o. gavage of corn oil, 5 days per week), albendazole (ABZ, p.o. gavage of 200 mg/kg ABZ in corn oil, 5 days per week), MMV665807 (p.o. gavage of 100 mg/kg MMV665807 in corn oil, 5 days per week). At the endpoint, mice were euthanized and parasite weight determined. The only effective treatment reducing parasite weight significantly was ABZ. (B) in a second experiment, mice were infected accordingly, and i.p. injection treatment started after 2 weeks of infection. The treatment groups of 8 randomly allocated mice were as follows: control (i.p. injection of DMSO three times per week and p.o. gavage of corn oil 5 days per week), ABZ (i.p. injection of DMSO three times per week and p.o. gavage of 200 mg/kg ABZ in corn oil 5 days per week), MMV665807 (i.p. injection of 100 mg/kg MMV665807 three times per week and p.o. gavage of corn oil 5 days per week) and MMV665807/ABZ (i.p. injection of 100 mg/kg MMV665807 three times per week and p.o. gavage of ABZ in corn oil 5 days per week). After euthanasia, parasite weight was determined. Effective were treatments of ABZ and MMV665807/ABZ. The drug MMV665807 did not lead to any reduction in parasite growth. P values shown in (A) and (B) were calculated by the non-parametric Wilcoxon signed-rank test with Bonferroni-adjustment.

In the first experiment, the effects of p.o. drug administration of MMV665807 were assessed in Balb/c mice i.p. infected with *E*. *multilocularis* metacestodes. The animals were treated 5 days per week by gavage of 100 mg/kg MMV665807 suspended in corn oil during 4 weeks. Mice treated with the standard drug ABZ (200 mg/kg, 5 days per week, 4 weeks) served as a treatment control. No adverse effects were observed during the treatment period. After 4 weeks, all mice were euthanized, metacestode tissue was carefully dissected and the parasite weight determined (Fig 7A). As the parasite weight data was not normally distributed according to Shapiro-Wilk test (W = 0.94; p = 0.18), non-parametric tests were chosen for further analysis. Kruskal-Wallis analysis showed that there was a significant difference between the groups (p = 0.005). Post-hoc analysis by Wilcoxon signed-rank test confirmed that oral treatment with MMV665807 had no effect on the parasite weight as compared to the untreated control. The orally applied control drug ABZ led to a significant reduction in parasite weight as compared to the control (p = 0.03), whereas administration of corn oil did not affect the parasite weight.

In a second experiment, MMV665807 was applied to *E*. *multilocularis*-infected mice by i.p. injection of 100 mg/kg MMV665807 three times per week for a period of 4 weeks. Mice treated with the standard drug ABZ (200 mg/kg, 5 days per week, 4 weeks) served as a treatment control. No adverse effects of any drug were noted. Assessments of the parasite weight after 4 weeks (Fig 7B) showed that this data set was not normally distributed (Shapiro-Wilk test: W = 0.88; p = 0.002) and therefore non-parametric tests were chosen for further analysis. Kruskal-Wallis analysis showed that there was a significant difference between the groups (p = 0.00003). Post-hoc analysis by Wilcoxon signed-rank test confirmed that i.p. treatment with MMV665807 did not result in a reduction of parasite weight, whereas in orally ABZ-treated animals, the parasite weight was significantly lower compared to the placebo group (p = 0.0009). A significant reduction was also observed for the MMV665807/ABZ double-treated group compared to the placebo group (p = 0.0009).

## Discussion

*E*. *multilocularis* causes the disease alveolar echinococcosis (AE) by the potentially unlimited growth of metacestodes in the liver of their intermediate hosts, and accidentally also in humans. These metacestodes consist of an outer acellular laminated layer, an inner germinal layer, and they are filled with vesicle fluid. The different cell types of the germinal layer include undifferentiated stem cells, glycogen storage cells, connective and muscle tissue, and nerve cells [27]. The parasite tissue produces metabolites that are continuously released, either to be integrated into the laminated layer, or to be further released to interact with the surrounding host tissue. Metabolites are also secreted into the vesicle fluid, where they potentially stimulate and/or influence the development and growth of the germinal layer of the parasite (for review see [7]). One of these components is the glycolytic enzyme PGI, a moonlighting protein that was shown to stimulate the proliferation of both the parasite germinal layer cells, as well as mammalian endothelial cells [28]. The fact that PGI is a prominent component of the vesicle fluid has been exploited for the development of the PGI screening assay [13], which allows the quantitative screening for drugs that impair the structural integrity of metacestodes in such a way that vesicle fluid, and hence PGI, is released into the medium supernatant. However, the PGI assay does not allow the detection of parasiticidal activity. Parasiticidal activity, however, is crucial for a candidate compound to be developed for the treatment of echinococcosis, because surviving stem cells will always be able to initiate the regrowth of metacestodes upon discontinuation of treatment, as it is the case for albendazole [29]. Thus, we elaborated an *in vitro* screening cascade that, for the first time, also includes the analyses of parasiticidal activity of a drug.

In a primary screen we tested all 400 Malaria Box compounds by applying the PGI assay, and in a second step we narrowed down the spectrum of active components by lowering the tested drug concentrations. This left us with the two potentially active compounds MMV665807 and MMV665794. Further, a therapeutic window was defined by including host IC_50_ value assessments on cultured parasite cells and mammalian cell cultures. This left MMV665807 as the most promising compound that could potentially be active *in vivo* without the risks of inducing detrimental effects on part of the host.

To test for parasiticidal activity *in vitro*, viability assays were adapted for the use in (i) cultures of isolated *E*. *multilocularis* germinal cells, and (ii) cultures of intact *E*. *multilocularis* metacestodes. In the germinal layer cell viability assay, MMV665807 exhibited an IC_50_ value of 0.6 μM, which was lower compared to what was observed in HFF and RH cell cultures. Mefloquine, known to be active against *E*. *multilocularis* metacestodes *in vitro* and *in vivo*, but not acting parasiticidal [16,17,19], was not nearly as active as MMV665807. The metacestode viability assay has the clear advantage that it provides a readout on the survival of the parasite tissue *in situ*, within the metacestode, and the results for MMV665807 (severe toxicity at 1.6 μM) are in agreement with our results obtained by TEM, where almost exclusively non-viable parasite tissue could be visualized, and clear effects also on the parasite stem cells have been noted. Again, in the vesicle viability assay mefloquine was active only at high concentrations (> 50 μM), which is in agreement with earlier studies that showed this compound does not act parasiticidal [16,17]. Despite the vesicle viability assay representing a very useful addition to the currently used screening methods, one cannot completely rule out that few single cells might still be viable after *in vitro* treatment. Nevertheless, compared to the PGI assay, the vesicle viability assay can give a much more reliable indication whether a drug could potentially act parasiticidal.

The screening of the Malaria Box, resulting in the identification of MMV665807, is a classic example of drug repurposing. Drug repurposing has been applied widely in the field of anthelminthics [14]. So far most of the drugs were obtained either from compounds applied in veterinary medicine or from drug libraries of potential anti-malarials. The reported uses of the Malaria Box include *in vitro* screening against *P*. *falciparum* early-stage gametocytes [30], schistosomula and adult worms of *Schistosoma mansoni*, as well as *in vivo* studies in *S*. *mansoni* infected mice [31]. Other applications include *Cryptosporidium parvum* [32], *Toxoplasma gondii* and *Entamoeba histolytica* [33], the oyster pathogen *Perkinsus marinus* [34], *P*. *falciparum* aminopeptidases [35], *Trypanosoma burcei* and *T*. *cruzi* [36], *Leishmania donovani* and *L*. *infantum* [36]. Interestingly, the most active compound against *E*. *multilocularis* metacestodes, MMV665807, was also amongst the 6 active compounds against *Perkinsus marinus*, but in that study selective toxicity assessments were not included [34].

Previous studies have reported on other anti-malarial drugs that exhibit activities against *Schistosoma* as well as *E*. *multilocularis*, including mefloquine [16,17,37] and artemisinin and derivatives [18,24,38]. However, with respect to the Malaria Box, there was no real overlap between the two species in terms of active compounds: for schistosomes, the one compound with most promising activity was MMV665852, with an *in vitro* IC_50_ against newly transformed schistosomula of 4.7 μM, and against adult worms of 0.8 μM, and promising *in vivo* activity (worm burden reduction of 52.5%, 46% and 31.2% for treatments at 400 mg/kg, 80 mg/kg and 4x100 mg/kg, respectively) [31]. The assessment of this compound against *E*. *multilocularis* metacestodes revealed only a marginal impact at 10 μM (14.8% PGI activity in medium supernatants) and no activity at all at 1 μM or lower concentrations. The second promising anti-*Schistosoma* compound, MMV007224 (IC_50_ against newly transformed schistosomula: > 33 μM; IC_50_ against adult worms: 0.8 μM) [31], was highly efficacious against *E*. *multilocularis* metacestodes at 10 μM, but lost its activity at 1 μM and was therefore not further characterized in this study. On the other hand, compounds MMV665807 and MMV665794 that were further tested within the present study against *E*. *multilocularis*, showed decent *in vitro* activities against *S*. *mansoni* (IC_50_ against newly transformed schistosomula of 1.8 and, > 33.3 μM, respectively; IC_50_ against adult worms of 0.6–1.3 and 1.1 μM, respectively [31]). However, these compounds were not further tested *in vivo* due to, as stated but not shown, unfavorable pharmacokinetic properties and toxicity.

Exposure of metacestodes to MMV665807 lead to high-level PGI release, loss of metacestode viability, and death of isolated germinal layer cells at concentrations that were several times lower than for mammalian HFF and RH cell cultures. Thus, this compound was further assessed in Balb/c mice secondarily infected with *E*. *multilocularis* metacestodes. P.o. application of the drug did not lead to a reduction of parasite mass. This is not surprising, since this compound exhibits rather poor pharmacokinetic properties: single dose p.o. application of 50 mg/kg results in a low plasma level of 0.06 μM (information provided through MMV in S3 Table. However, this data is based on a single p.o. dose, and only blood levels were measured. There is no information on the level of MMV665807 or putative metabolic products in other tissues. In our experiments, a long-term treatment of *E*. *multilocularis* infected mice was performed. I.p injection was applied in order to potentially overcome the bias of low drug uptake, but this did not yield any improvement in treatment efficacy. This demonstrates that the compound is not only poorly absorbed, but also does not reach its target(s) in the parasite, possible due to rapid metabolic breakdown and/or other unfavorable pharmacodynamic properties. These aspects should be further investigated, but were not within the focus of this this study.

MMV665807 is a salicylanilide-derivative, similar to the already commercially available drug niclosamide, an anti-cestode drug [39] that is used against adult stages of cestodes. Niclosamide is known to be hardly soluble and poorly absorbed, leading to low bioavailability. Niclosamide has gained renewed attention recently in the possible treatment of breast cancer, since it kills breast cancer cells *in vivo* and *in vitro* [40]. As other parasites, *E*. *multilocularis* metacestodes share many growth characteristics with malignant tumors [41], and anti-cancer drugs have been suggested for repurposing for the treatment of AE [6]. Niclosamide was also shown to exhibit pronounced *in vitro* activity against apicomplexan parasites such as *T*. *gondii* [42]. A series of niclosamide derivatives (including MMV665807) were also assessed against *T*. *gondii*, but MMV665807 exhibited only moderate *in vitro* activity and was not further assessed *in vivo*. Lee et al analyzed the structure activity relationship of 94 salicylanilides against *Mycobacterium tuberculosis* and macrophage host cells [43]. Amongst those, MMV665807 exhibited activity against *M*. *tuberculosis* (MIC = 3.1–6.3 μM), but was also active against murine macrophages (IC_50_ = 5.9 μM) [43], thus leaving no therapeutic window. As a mode of action Lee et al suggested destruction of the cellular proton gradient. Other compounds with structures closely related to MMV665807 showed activity against transmembrane protease serine 4 [44]. However, up to date none of these drug targets have been experimentally proven for MMV665807.

In conclusion, *in vitro* screening of the Malaria Box resulted in the identification of MMV665807 as a potential drug candidate for the treatment of *E*. *multilocularis* infections with a pronounced selective anti-parasitic toxicity *in vitro*. However, these effects could not be reproduced *in vivo*, most likely due to the fact that MMV665807 is poorly absorbed and exhibits unfavorable pharmacodynamic properties, which need to be improved. MMV665807 derivatives that retain selective toxicity, but exhibit improved bioavailability, could hold a significant potential as treatment option for AE and also CE.

## Supporting Information

S1 Table Malaria Box screening by PGI assay. (XLS)

S2 Table AlamarBlue vesicle viability assay values and SDs corresponding to Fig 3. (XLSX)

S3 Table Pharmacokinetic information on MMV665807 as provided by MMV. (XLS)
